# Detection and characterization of bovine viral diarrhea virus in beef cattle imported from Australia to West Java, Indonesia

**DOI:** 10.14202/vetworld.2023.1468-1476

**Published:** 2023-07-19

**Authors:** Aditya Primawidyawan, Surachmi Setiyaningsih, Retno Wulansari, Mawar Subangkit, Bambang Pontjo Priosoeryanto

**Affiliations:** 1 Doctoral Study Program in Animal Biomedical Sciences, School of Veterinary Medicine and Biomedical Sciences, IPB University, Bogor, Indonesia; 2 Division of Veterinary Microbiology, School of Veterinary Medicine and Biomedical Sciences, IPB University, Bogor, Indonesia; 3 Division of Veterinary Internal Medicine, School of Veterinary Medicine and Biomedical Sciences, IPB University, Bogor, Indonesia; 4 Division of Veterinary Pathology, School of Veterinary Medicine and Biomedical Sciences, IPB University, Bogor, Indonesia

**Keywords:** bovine viral diarrhea virus, clinical symptoms, detection, imported cattle, West Java

## Abstract

**Background and Aim:**

To meet domestic demand for meat, Indonesia imports live cattle from Australia, which have non-bovine viral diarrhea (BVD)-free status. The consequence of importing live cattle from Australia is potentially introducing a novel BVD variant to Indonesia. Therefore, detecting BVD early and determining the agent’s characteristics and clinical symptoms are necessary. This study aimed to detect and characterize clinical symptoms of bovine viral diarrhea (BVD) and highlight the importance of farm management as a risk factor for the spread of BVD. This study aimed to provide information about the effectiveness of preventive measures against BVD in Australian-imported cattle at the Tanjung Priok Seaport Agricultural Quarantine. Bovine viral diarrhea is among the most common diarrheal diseases found in feedlots and is a severe health and economic problem in cattle.

**Materials and Methods:**

All cattle in a selected feedlot were examined for clinical symptoms on their first day of arrival. The sampling criteria included age, body weight, body temperature (BT), animal breath (AB), pulse (PL), conjunctivitis (CJ), hyperlacrimation (HL), hypersalivation (HS), DR, fever, limping leg (LL), emaciation, stomatitis (ST), weakness (WK), and coronitis (CR). In addition, 64 blood samples were taken from cattle that exhibited clinical symptoms of BVD. On the 3^rd^ day of arrival, a blood sample showing positive clinical symptoms was examined using antigen (Ag)-capture enzyme-linked immunosorbent assay (ELISA). The data from these clinical symptoms were analyzed alongside the laboratory results using multidimensional scale analysis, heatmap distribution, and principal component analysis (PCA). Furthermore, the positive serum samples obtained from the Ag-capture ELISA underwent a nested multiplex polymerase chain reaction and molecular detection and genetic characterization of BVDV based on the 5’ untranslated region of the viral genome, followed by sequence and phylogenetic tree analyses.

**Results:**

Using PCA, 12 clinical symptom characteristics of BVD were determined from 13 clinical symptoms synergized with five cattle positive for Ag-capture ELISA. The clinical symptoms included internal factors such as physiological conditions of CJ, HL, HS, DR, BT, LL, loss of appetite, ST, WK, CR, AB, and PL. The screening test showed that five samples tested positive for the BVD Ag, while 59 tested negative. Phylogenetic tree analysis using a 360-nucleotide portion of the NS5B gene showed that Sample B23F5R had a distinct path compared to the other two samples in the phylogenetic diagram. The profile of sample B23F5R was closely related to BVDV reference subgenotype 1-a group (NCBI, access no. LC068605), with a homology percentage of 92.36%. Furthermore, this sample was similar to the BVDV reference 1-a, Strain 12, identified in Japan. The other two samples, B13F5R and A13F5R, showed close resemblance to the BVDV reference subgenotype 1-a that had been previously identified in Indonesia (NCBI, access no. MK411755), with homology percentages of 97.81% and 97.75%, respectively.

**Conclusion:**

The BVDV-1a strain is the main subtype present in beef cattle imported from Australia to West Java, Indonesia. The characteristics of clinical symptoms associated with BVD infection comprised 12 symptoms synergized with the positive sample in the PCA. The present results can facilitate the development of preventive and control measures for BVD circulation in Indonesia.

## Introduction

Bovine viral diarrhea virus (BVDV) is widely recognized as having a significant economic impact on infected livestock. This disease is characterized by animals with persistent infection (PI), reproductive disorders, decreased production, poor growth, weakness (WK), and an increased incidence of other diseases. Bovine viral diarrhea virus is caused by a positive-sense single-stranded ribonucleic acid (RNA) virus belonging to the genus of *Pestivirus* within the *Flaviviridae* family [1, 2]. *Pestivirus* comprises four species: Bovine viral diarrhea virus Type 1 (BVDV-1, *Pestivirus* A), BVDV Type 2 (BVDV-2, *Pestivirus* B), classical swine fever virus (*Pestivirus* C), and border disease virus (*Pestivirus* D) [3–5]. Recent studies by Rivas *et al*. [5] and Sozzi *et al*. [6] have also identified new viruses that may belong to the genus *Pestivirus*. Through nucleotide sequence comparisons, 21 BVDV-1 subgenotypes (BVDV-1a to BVDV-1u) and four BVDV-2 subgenotypes (BVDV-2a to BVDV-2d) have been identified [4, 7]. The first case of BVD in Indonesia was reported in Bali cattle in Sulawesi in 1989 [8], and there were no further reports until 2009. In 2011, approximately 43.2% of beef, dairy, and breeding cattle were found to be seropositive for BVDV [9], increasing to 46% in 2013 [10]. According to another study, the high seroprevalence of BVDV significantly damages beef industry production [9]. Previous genotyping study on BVDV associated with diarrhea and respiratory disorders in Indonesia detected the -1a to -1d subgenotypes based on the 1-a dominant NS5B gene [10]. With a population of 260 million people, Indonesia requires significant animal protein, and meeting this demand requires livestock, particularly beef cattle, and capable of producing high-quantity and high-quality products [11]. Domestic meat production, including cattle production, cannot currently meet the animal protein needs of the Indonesian people; therefore, Indonesia imports meat and live beef cattle from countries such as Australia, reported as a non-BVD-free country. The presence of BVDV in cattle farms threaten beef cattle productivity, specifically weight gain. In Australia, BVDV serotypes 1-a, 1-b, and 1-c are classified as the second most economically significant livestock disease after parasitic tick infections and as the most important pathogen in tick-free areas [12]. Bovine viral diarrhea causes an estimated loss of $114 million (AUD) annually in Australia [13].

Laboratory examinations are essential for the early detection of animal diseases and to maximize preventive measures. However, the difficulty in diagnosing several diseases, including BVD, often hinders early detection. The differential diagnosis of BVD includes diseases such as Rotavirus infection, malignant catarrhal fever, Infectious Bovine Rhinotracheitis, enzootic bovine leukosis, and rinderpest or foot and mouth diseases. Conventional diagnosis of BVD has been conducted using Ag-capture ELISA procedures, followed by nested multiplex polymerase chain reaction (PCR) tests [11, 14, 15].

Importing live cattle from Australia presents the risk of introducing new BVDV serotypes to Indonesia. Therefore, comprehensive detection and identification of the BVD agent are crucial. In this study, we aimed to conduct diagnoses based on clinical symptom characteristics and identify the BVD agent following the roadmap of animal disease prevention and control in Indonesia.

## Materials and Methods

### Ethical approval

The present study was performed after approval from the Ethical Research Committee, School of Veterinary Medicine and Biomedical Sciences, IPB University, Bogor, Indonesia (No. 041/KEH/SKE/X/2022).

### Study period and location

The study was conducted from January to September 2022. Imported Brahman Cross beef cattle from Australia, aged >2 years with the clinical symptoms were taken as a sample from local farmers in areas of Sukabumi, Bogor, Tangerang, Cianjur, and Subang.

### Clinical symptoms and blood collection

#### Clinical symptoms

On the 1^st^ day of arrival at the selected feedlot, all cattle were examined for clinical symptoms. The sampling criteria included age, body weight (BW), body temperature (BT), animal breath (AB), pulse (PL), conjunctivitis (CJ), hyperlacrimation (HL), hypersalivation (HS), DR, fever, limping leg (LL), emaciation, stomatitis (ST), WK, and coronitis (CR). The normal parameter values for cattle are temperature between 36°C and 39°C, breathing rate between 15 and 30 breaths/min, PL rate between 60 and 80 beats/min, and BW exceeding 355 kg [10, 13]. Other clinical symptom parameters are assessed based on the severity of symptoms using a scoring system where 0 indicates no symptoms, 1 indicates mild symptoms, 2 indicates moderate symptoms, and 3 indicates severe symptoms. The data from these clinical symptoms were analyzed along with the laboratory results through multidimensional scale analysis, heatmap distribution, and principal component analysis (PCA). Cattle with positive clinical symptoms were isolated, observed, and assessed based on the condition of the observed symptoms.

#### Blood collection

Blood samples were obtained from cattle with positive clinical symptoms on the 3^rd^ day of arrival. Approximately 3 mL of blood was drawn from the coccygeal vein using an ordinary vacutainer tube (Becton Dickinson, NJ, USA). Subsequently, the whole blood samples were processed through centrifugation at 1500*× g* for 15 min to collect serum, which was subsequently stored in a freezer at −20°C until further use for molecular detection and genetic characterization of BVDV.

### Bovine viral diarrhea screening using Ag-capture ELISA

The Ag-capture ELISA was conducted using the BVDV Ag ELISA Kit (IDEXX® USA) to detect the Erns Ag of BVDV. The materials used included the BVD Ag ELISA Kit (IDEXX), methanol (Merck no. 1.00983.2500), 20 mM tris-HCL (Merck no. 1.08382.0100), distilled water, tween 20 (Merck No. 8.22184.0500), and 67 mM phosphate buffer pH 7.2. The ELISA procedure for BVD was conducted on the serum samples following the manufacturer’s recommendation. The BVD Ag Kit was incubated at 18°C–26°C for 1 h. Subsequently, each well of the test plate was filled with 100 μL sample diluents. Four wells in the first microplate column were emptied of serum to serve as positive and negative control wells. A total of 25 μL of negative control was added to wells A1 and B1, while 25 μL of positive control was added to wells C1 and D1. The sample serum was added to well E1 and subsequent wells according to the established pattern (25 μL). Afterward, the plate was shaken and incubated for 90 min at 18°C–26°C. Washing was performed using 300 μL of washing solution, and aspiration was conducted five times until the solution touched the well walls. A total of 100 μL of conjugate reagent was added to each well and incubated for 30 min at 18°C–26°C, followed by another washing step. Up to 100 μL of Tetra Methyl Benzidine substrate was added to each well, and then the microplate was covered with aluminum foil and incubated for 10 min at room temperature (18°C–26°C) in a dark room. Subsequently, 100 μL of stop solution was added to stop the reaction, and the absorbance was measured using an ELISA reader at a wavelength of 450 nm. Using a spectrophotometer, the absorbance results were measured and recorded by setting several protocols (using Bio-Tek’s K junior protocol) [10, 16]. The presence or absence of BVD Ag in the sample was determined by calculating the numerical optical density value called S-N (sample value minus negative control value). The OD value in the sample is reduced by the average value of the negative control. A positive sample was identified from the Ag ELISA when the S-N value was >0.3 and accompanied by corresponding clinical symptoms. The observed positive samples were documented in a prepared questionnaire [10, 16].

### Synergistic analysis

Synergistic analysis uses multivariate analysis (MVA), which is based on the principles of multivariate statistics. Typically, MVA is employed when multiple measurements are made on each experimental unit, and the relationships among these measurements and their structures are important. A modern, overlapping categorization of MVA includes normal and general multivariate models, distribution theory, the study and measurement of relationships, probability computations of multidimensional regions, and the exploration of data structures and patterns [17].

There are different models, each with its own type of analysis:

The distance calculation between parameters was calculated using Z-score with the formula as follow:

Z-score = (x – m)/SD

x = data point, μ = mean, SD = standard deviation.

### Multidimensional Scale

The correlation and group clustering analyses between the samples with positive clinical symptoms and positive Ag-capture ELISA results were conducted using a multidimensional scale graph in an R software plot (www.r-project.org). The parameter distance was calculated using the Euclidian method (www.r-project.org) [18, 19].

### Heatmap Distribution

Using the heatmap distribution method, the severity of the clinical symptoms in each positive cattle was analyzed with G-plot package computer software (www.r-project.org) utilizing a Z-score analysis of the clinical symptom data. The Z-score levels were measured statistically using R software with the G-plot package. The heatmap, color-coded from blue to red to indicate different levels of severity, showed the gradation of clinical symptom severity and the grouping of parameters based on homology level (www.r-project.org) [18, 19].

### Principal component analysis

The relationship between positive clinical symptom samples and serological positivity was analyzed through PCA using R software(www.r-project.org). Principal component analysis was conducted in R software using the FactoMineR package. The clinical data pattern was distributed in four quadrants using two dimensions (Dim^1^ and Dim^2^). A high correlation (positive or negative) was indicated by the close direction of the arrows [18, 19].

### Ribonucleic acid isolation and nested multiplex PCR

Viral RNA was extracted from serum samples using a Viral Nucleic Acid Kit II (Geneaid^®^, Geneaid Biotech Ltd., Taiwan) following the manufacturer’s instructions. The extracted RNA was subjected to a nested multiplex PCR BVD assay, followed by an agarose gel resolution process through electrophoresis to determine the BVDV genotype at 360 bp (BVD 1) or 612 bp (BVD 2). The specific NS5B primers used for the external reactions and nested multiplex genotypes are listed in Table-1 [11, 16]. The first step of the external reverse transcription (RT) reaction was conducted using the RT/platinum RT-PCR Kit (Invitrogen, Carlsbad, USA) as specified by the manufacturer. The thermal conditions included 30 min of RT at 37°C, an initial denaturation for 3 min at 94°C, followed by 25 cycles each for 20 s at 94°C, 30 s at 50°C, 30 s at 72°C, and a final elongation for 15 min at 72°C. The reaction was then held at a constant temperature of 4°C. The second stage of multiplex PCR genotyping was conducted using the Kapa2G Fast ReadyMix Kit (Qiagen^®^, Hilden, Germany). The reaction was heated to 95°C for 3 min, followed by 35 cycles at 94°C for 15 s, 50°C for 15 s, 72°C for 15 s, and a final elongation at 72°C for 5 min. The reaction was held at a constant temperature of 4°C. The Ag-capture ELISA was performed using the BVDV Ag ELISA Kit (IDEXX^®^) to detect the Erns Ag of BVDV. This Ag-capture ELISA was considered a screening test for cattle with PI. The BVDV-1 Singer strain, produced by the Indonesian Research Institute of Veterinary Science, was used as a positive control for genotype-1. All PCR products were separated on a 1.5% agarose gel, stained with SYBR® Safe DNA gel, and visualized using the gel documentation system from Bio-Rad, (California, USA) [20, 21].

**Table-1:** Primer sequences for detection and genotyping in nested PCR BVD [11].

Primer	Sekuen (5’-3’)	Position on the NS5B gene	PCR product size
External forward	AAGATCCACCCTTATGA(A/G)GC	10385–10404	1100 bp
External reverse	AAGAAGCCATCATC(A/C)CCACA	11528–11547	
Internal forward 1	TGGAGATCTTTCACACAATAGC	10758–10779	360 bp (BVDV-1)
Internal forward 2	GGGAACCTAAGAACTAAATC	10514–10533	604 bp (BVDV-2)
Internal_reverse	GCTGTTTCACCCAGTT(A/G)TCAT	11096–11117	

BVD=Bovine viral diarrhea, PCR=Polymerase chain reaction

### Sequencing and phylogenetic tree analyses

Sequencing was conducted on the sample that tested positive for the second stage nested multiplex PCR to confirm the BVDV genotype based on the partial NS5B gene. Using proportional calculations, we selected three samples with positive nested multiplex PCR results from five feedlot location as representative samples to eliminate bias. The selected PCR products were sequenced at 1^st^ BASE, Malaysia. Thepartial NS5B gene sequence analysis was conducted using MEGA version 6 (Megasoftware, USA). The nucleotide sequences of three known BVDV strain samples obtained from GenBank were aligned using the MUSCLE algorithm. Sequencing was conducted using ABI PRISM sequencer engine 3130 models and the Big Dye Terminator v3.1. Cycle Sequencing Kit (Part No. 4337455, Applied Biosystems, USA) following the manufacturer’s recommendations. Primer pairs (forward and reverse) with a 5–10 pmol/mL concentration were used. A DNA concentration of 3–10 ng/mL with a total volume of 20 mL was required for sequencing [10]. The identities of the RT-PCR products obtained from the animal serum samples were confirmed directly using specific forward and reverse primers. The obtained sequences were compared with those stored in GenBank using the BLAST software (http://blast.ncbi.nlm.nih.gov/Blast.cgi). The sequences derived from this study will be stored in GenBank using the MEGA v4.1 program. The evolutionary distance was calculated by constructing a phylogenetic tree based on the 360 nt 5’ untranslated region using the maximum likelihood method and a bootstrap value of 1000× with MEGA-X. The degree of similarity between the nucleotide sequences was determined using the BioEdit software v.8.0 [20, 22].

## Results

### Clinical symptoms

**Figure-1:**
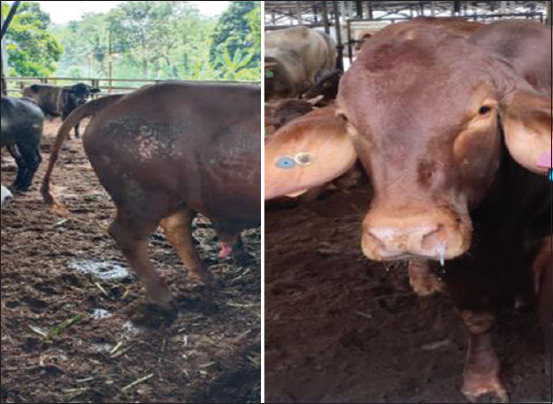
Most common clinical symptom of bovine viral diarrhea (Diarrhea and Hypersalivation) in Cattle.

Based on the clinical symptoms, 64 positive samples (12.3%) were identified from 520 samples. The positive samples comprised 11 samples from Sukabumi, 10 from Bogor, 13 from Tangerang, 16 from Cianjur, and 14 from Subang (Figure-1).

### Antigen-capture ELISA

Using the Ag ELISA, five positive samples (7.8%) were observed from the 64 samples and were distributed as follows: Two samples from Sukabumi, one from Bogor, one from Tangerang, and one from Subang. The OD values varied among the samples, ranging from 0.545 to 2.191, while the S-N values ranged from 0.534 to 2.080, with the maximum value observed in the Sukabumi samples. The detailed Ag-capture ELISA results for each positive sample are shown in Table-2.

**Table-2:** BVD serological test results with antigen capture ELISA.

Area	Number of samples	Total samples	Positive	Negative	Opacity density samples	Positive sample code and S-N	Positive percentage
Sukabumi	11	100	2	9	A13F5R (1.211) and B13F5R (2.191)	A13F5R (1.100) and B13F5R (2.080)	3.125
Bogor	10	100	1	9	B23F5R (1.180)	B23F5R (1.069)	1.56
Tangerang	13	100	1	12	C13F5R8*(0.741)	C13F5R*(0.630)	1.56
Subang	14	100	1	13	D13F5R*(0.545)	D13F5R*(0.534)	1.56
Cianjur	16	120	0	16	---	---	0
Total	64	520	5	59	---	---	7.8

* The PCR result was negative because the OD ELISA value of the antigen sample was small value. BVD=Bovine viral diarrhea, PCR=Polymerase chain reaction, ELISA=Enzyme-linked immunosorbent assay, OD=Opacity density

### Synergistic analysis

#### Multidimensional scale

**Figure-2:**
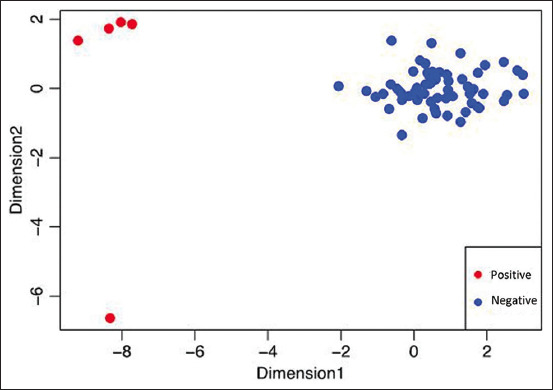
Data analysis using the Multidimensional Scale method regarding descriptive data on clinical symptoms on sample positivity. (Blue color is a clinical symptom in a sample that has a match with a positive bovine viral diarrhea [BVD] sample value and red color is a clinical symptom that does not match the BVD positive sample value).

The results of the multidimensional scale analysis comparing clinical symptoms with the Ag-capture ELISA show a significant distinction between the positive (red) and negative (blue) samples. The five samples with positive Ag-capture ELISA results appeared to be perfectly clustered compared to the remaining 59 samples with negative Ag-capture ELISA results (Figure-2).

#### Heatmap distribution

**Figure-3:**
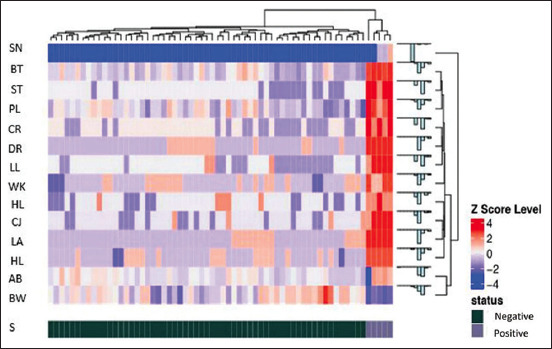
Data analysis using the Heatmap Distribution method regarding descriptive data on clinical symptoms on sample positivity. (A=S-N, BT=Body temperature, ST=Stomatitis, PL=Pulse, CR=Coronitis, DR=Diarrhea, LL=Limping leg, WK=Weakness, HL=Hyperlacrimation, CJ=Conjungtivitis, LA=Loss appetite, HL=Hypersalivation, AB=Animal breath, BW=Body weight and S=Status).

The five samples that tested positive for the Ag-capture ELISA results and exhibited positive clinical symptoms had the highest Z-score level, indicated by a strong red color. Conversely, the 59 samples that tested negative for Ag-capture ELISA samples but showed positive clinical symptoms exhibited various colors, indicating varying degrees of severity in their clinical symptoms (Figure-3).

#### Principal component analysis

**Figure-4:**
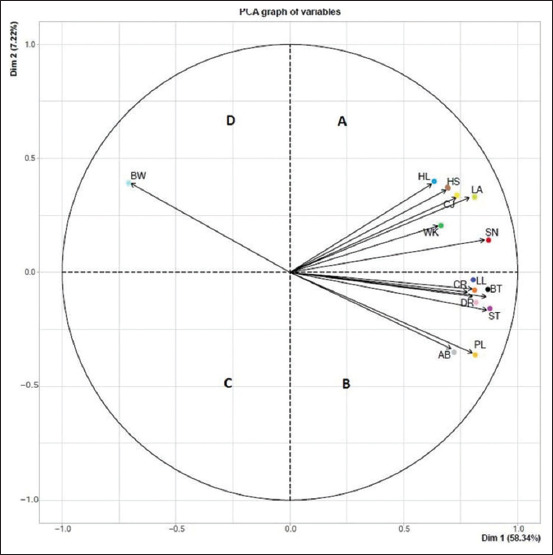
Principal Component Analysis method data analysis regarding the synergy of clinical symptoms on sample positivity. (Quadrant A: S-N (red), WK/green, CJ/lime, LA/yellow, HL/turqoise, HS/brown (Quadrant B: CR/orange, LL/indigo, BT/black, DR/rose, ST/purple, PL/gold, and AB/grey, (Quadrant D: BW/aqua). WK=Weakness, CJ=Conjunctivitis, LA=Loss appetite, HL=Hyperlacrimation, HS=Hypersalivation, CR=Coronitis, LL=Limping leg, BT=Body temperature, DR=Diarrhea, ST=Stomatitis, PL=Pulse, AB=Animal breath, BW=Body weight.

The PCA showed four quadrants. Body weight was isolated in one quadrant in the diagram, indicating its poor correlation with the other clinical symptom parameters. Five of the 13 clinical symptoms showed a high positive correlation with S-N, which serves as a benchmark for BVD infection screening (Figure-4). Among these five, WK showed the highest positive correlation with S-N, followed by CJ, loss of appetite (LA), HS, and HL; all these symptoms were located in quadrant A. Conversely, the remaining seven (LL, DR, CR, BT, ST, PL, and AB) clinical symptoms were located in the quadrant B of S-N. In quadrant D, BW occupied the most distant position, indicating the weakest associated with the clinical symptoms observed in the study.

### Polymerase chain reaction analysis of Ag-capture ELISA-positive samples

**Figure-5:**
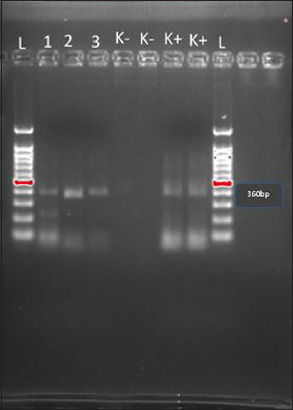
Reverse transcription-polymerase chain reaction analysis of samples for the 5’ untranslated region. Lane L=100 bp DNA marker, Lane 1=bovine viral diarrhea (BVD) virus positive samples (A13F5R), Lane 2=BVD virus positive samples (B13F5R), Lane 3=BVD virus positive samples (B23F5R), Lane K-=Negative control, and Lane K+=Positive control.

The PCR analysis of the five samples with positive Ag-capture ELISA results showed that three samples (60%) had a strong band of 360 bp, while the remaining two had a weak signal Figure-5. The electrophoresis results indicated that samples 1 (A13F5R), 2 (B13F5R), and 3 (B23F5R) showed line conformity, even though the line appeared thinner than that formed in the positive control. The observed line corresponded to a marker around 360 bp, consistent with the BVDV-1 group, which has a characteristic wave around 360 bp. The specific primers used in the nested multiplex PCR amplification targeted the internal stage and produced a 360 bp amplicon for the BVDV-1 genotype, while a 604 bp amplicon was generated for the BVDV-2 genotype. The test results showed that all three samples belonged to the BVDV-1 genotype, as evidenced by a single DNA band forming at 360 bp (Figure-5).

### Sequence and phylogenetic tree analyses

**Figure-6:**
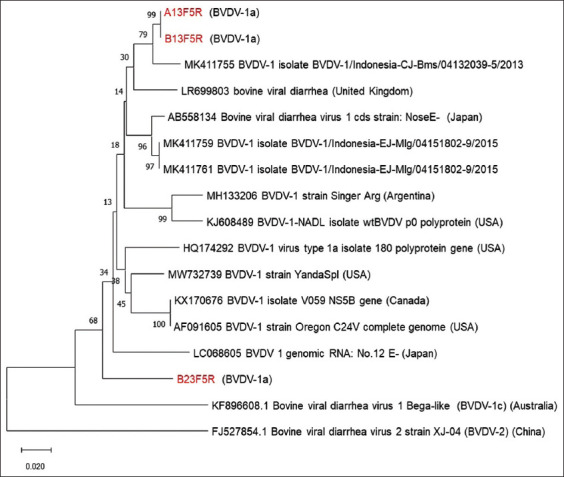
Phylogenetic tree based on the 5’UTR region along 360 nt with Maximum Likelihood method and bootstrap number 1000× using MEGA-X (The sample code colored red is the research sample).

Result of sequencing and phylogenetic tree analysis indicated that only three samples (A13F5R and B13F5R from Sukabumi and B23F5R from Bogor) were positive for BVDV. The sequencing data showed that the profile of Sample B23F5R was closely related to the BVDV reference 1-a group (NCBI, access no. LC068605), with a homology percentage of 92.36%. In addition, this sample was similar to the BVDV reference 1-a, Strain 12, reported in Japan. Conversely, B13F5R and A13F5R were closely similar to the BVDV reference subgenotype 1-a previously identified in Indonesia (NCBI, access no MK411755), with homology percentages of 97.81% and 97.75%, respectively (Figure-6).

## Discussion

The literature on clinical symptoms associated with BVD shows various manifestations, including varying levels of fever, anorexia, lethargy, leukopenia, ocular and nasal discharge, oral erosions and ulcers, oral papilla blunting and hemorrhage, DR, depression, pneumonia, decreased BW, bloat, tachypnea, and epithelial erosions in various areas such as the interdigital space, coronet, teats, or vulva [23] In this study, we observed 13 clinical symptoms correlated with Ag-capture ELISA results, including CJ, HL, HS, DR, BT (fever), LL, LA, ST, WK, CR, PL, decreased BW, and AB. However, the relationship was weak. The observed clinical symptoms (13) accounted for only 76% of the total clinical symptoms (17) reported in the literature; only about 76% were observed in livestock in this study, indicating that clinical symptoms alone are insufficient for BVD diagnosis. This was confirmed by the data from all analyzed samples, where only 64 (approximately 12.30%) out of 520 total samples exhibited the observed clinical symptoms. Similar findings were reported by Saepulloh and Sendow [11], who identified 69 positive BVD cases (11.74%) in the West Java area out of 588 total examined samples. When comparing the samples that tested positive for Ag-capture ELISA to the total samples with positive clinical symptoms of BVD, only five out of 64 samples (7.8%) tested positive for the Ag-capture ELISA, and only three of these samples were successfully sequenced. The Sukabumi area had the highest number of positive samples, with two samples, followed by the Bogor, Tangerang, and Subang areas, with one positive sample each. Saepulloh and Sendow [11] also reported positive cases in the West Java area, specifically in the Bogor, Sukabumi, Sumedang, and Bandung areas. They successfully sequenced seven positive samples (10.1%) out of 69 total samples found.

Several studies have proven that BVDV transmission occurs horizontally and vertically [24, 25]. In a previous study, blood samples from 400 animals were tested, and Ag-positive individuals were retested at 21-d intervals to identify animals with PI, although 1.27% (3/236) of the heifers and cows were identified as acutely infected, no PI was detected among the animals [24]. Calves were screened using blood samples either in the precolostral stage or after 3 months of age, and the rate of persistently infected calves born in the herd was 4.84% (8/164). Due to the urgent elimination of persistently infected calves and the implementation of a vaccination schedule, no hatchling with PI was identified at the next calving period [24]. Almeida *et al*. [25] emphasized the role of artificial insemination technicians in transmitting the virus through contaminated shoes, clothing, equipment, and semen. The pathogenesis of BVD and the resulting clinical symptoms are influenced by several factors, including biotype, viral virulence, host immune status, host physiological status, and environmental conditions. Bovine viral diarrhea virus infection contributes to the occurrence of infectious diseases in cattle, often occurring in conjunction with other pathogens in field cases. A salmonellosis outbreak in dairy cattle was associated with the presence of cattle with PI, inducing immunosuppression in other cows.

The ratio of samples that tested positive for Ag-capture ELISA to the total samples with positive clinical symptoms was low, with only 7.8% (5/64) of samples testing positive. This result can be attributed to the high sensitivity and specificity of the Ag-capture ELISA, which makes it reliable. Bovine viral diarrhea diagnostic Kit tests can be used to detect BVDV based on BVDV-specific Ags or antibodies. Antigen-capture ELISA provides a simple and fast method for detecting persistently infected animals, which is ideal in a large animal population. Therefore, BVD detection in herd populations can be effectively conducted. The sensitivity and specificity of the Ag-capture ELISA reportedly range from 97% to 100% for sensitivity and 98.8%–100% for specificity compared to virus isolation [26]. The Ag-capture ELISA has been developed for use with various samples, such as serum, milk, and ear notches. The Ag-capture ELISA is a simple and cost-effective diagnostic method with several advantages, including its powerful sensitivity and specificity; furthermore, it does not require cell culture facilities, and test results are minimally affected by prolonged storage [26, 27]. Ag-capture ELISA is a sensitive and reliable serological technique used regularly for detecting PI of animal diseases, including BVD infection. Moreover, it is simpler than nested multiplex PCR [26, 27].

A total of 13 clinical symptoms showed a significant relationship with the S-N graph data, which served as the main parameter for determining positivity. The PCA analysis divided the symptoms into quadrants A, B, C, and D. These quadrants demonstrate that clinical symptoms within the same area of positive value (S-N) tend to have a closer and stronger relationship. Quadrant A comprises five clinical symptoms (WK, CJ, LA, HL, and HS) strongly correlated with positive values (S-N). These clinical symptoms indicate that a positive value (S-N) is strongly influenced or accompanied by dominant clinical symptoms. The synergy PCA analysis shows that WK, CJ, and LA were the dominant clinical symptoms in cattle, leading to depression. This finding agrees with Lanyon’s study [27], which highlighted these symptoms as predominant in Australian cattle. Weakness, CJ, and LA are symptoms that affect cows in the early phase, followed by conditions that worsen with HS and HL. Clinically, acute BVDV infections can vary from subclinical to fatal, with commonly reported clinical signs including pyrexia, intermittent DR, nasal and ocular discharge, depression, and slight dry cough. Acute BVDV infections have been reported to be immunosuppressive, increasing susceptibility to secondary infections and worsening clinical disease severity [27]. Similarly, Everman and Barrington [28] identified acute symptoms, including fever, leukopenia, depression, anorexia, ocular and nasal discharge, oral lesions, and DR, which supports the clinical symptom findings in this study. Quadrant B comprises seven clinical symptoms (LL, DR, CR, BT, ST, PL, and AB). These symptoms occupy different positions in the quadrant relative to a positive value (S-N), indicating a weaker synergy with BVD positive results. A clinical symptom of BW is positioned furthest in quadrant D, indicating the weakest interpretation of clinical symptom among the clinical symptoms observed in the study.

The phylogenetic diagram shows that Sample B23F5R showed a distinct divergence from the other two samples. Its profile in the NCBI application was closely related to BVDV strain 1a, with a 92.36% similarity to profile LC068605, which corresponds to BVDV reference 1-a, Strain 12, found in Japan. The clinical symptoms reported by studies on the reference 1-a virus in Japan included poor development, DR, respiratory symptoms, and mucosal disease [29]. The clinical symptoms observed in our study were similar to those reported by studies on the reference 1-a virus in Japanese cattle, such as emaciation, fever, DR, and respiratory symptoms (HL and HS). These findings indicate that this virus serotype tends to induce general symptoms, with developmental disorders in livestock being a key aspect.

The B13F5R and A13F5R samples showed proximity to BVDV subgenotype 1-a identified in Indonesia (NCBI, access no. MK411755), with percentages of 97.81% and 97.75%, respectively. In a previous study by Irianingsih *et al*. [10] on access number MK411755, the clinical symptom criteria for BVD reference 1-a were observed in East and Central Java dairy cattle. These criteria included fever, emaciation, DR, and respiratory problems. These clinical symptoms are similar to those observed in our study, even though the research object uses dairy cows. Irianingsih *et al*. [10] stated that the potential spread of BVDV serotype 1-a in Indonesia was due to the importation of cattle from abroad, as the evidence obtained showed that the serum examined from 2013 to 2021 had a phylogenetic closeness and sufficient resemblance to the Australian virus isolate [10, 30]. At present, the available BVDV tests in Indonesia for imported cattle involve Ag- and antibody-capture ELISA. However, semen tests to detect PI are not conducted. Furthermore, only a small proportion of feeders are routinely screened for BVDV Ag in quarantine. Due to the low prevalence of PI, sampling techniques may not be sensitive enough to detect and remove all persistently or transiently infected individuals from imported cattle. Therefore, testing of all animals is required. High seroprevalence of BVDV has been reported in the imported feeder and finisher cattle, and Ags have been detected in serum samples of these animals in quarantine facilities [30].

## Conclusion

The BVDV-1a is the main subtype present in beef cattle imported from Australia to the West Java region in Indonesia. This result is consistent with previous results in domestic cattle in Indonesia. The reported results can facilitate the design of preventive and control measures for BVD circulation in Indonesia. However, future studies with further samples and an extended study period are required to better evaluate the interrelationships between BVDV infection and clinical symptoms in beef cattle imported from Australia.

## Authors’ Contributions

AP: Blood sampling and nested multiplex PCR analysis. SS and MS: Sequencing and phylogenetic tree analyses. RW: Clinical signs analysis. BPP: Designed and supervised the experiments. All authors participated equally in preparing the manuscript for publication. All authors have read and approved the final manuscript.
